# Supplementary material for: Chromatin accessibility promotes hematopoietic and leukemia stem cell activity
Source: Nat Commun. 2020 Mar 16;11:1406. doi: 10.1038/s41467-020-15221-z (PMC7076002; doi:10.1038/s41467-020-15221-z)
Supplement: Supplementary file 1 — Supplementary Information [file 41467_2020_15221_MOESM1_ESM.pdf]

Supplementary Information

Chromatin accessibility promotes hematopoietic and leukemia stem cell activity

Lucia Cabal-Hierro[1,2], Peter van Galen[2,3], Miguel A. Prado[4], Kelly J. Higby[1,2], Katsuhiro Togami[1,2], Cody T. Mowery[1,2], Joao A. Paulo[4], Yingtian Xie[1,5], Paloma Cejas[1,5], Takashi Furusawa[6], Michael Bustin[6], Henry W. Long[1,5], David B. Sykes[7], Steven P. Gygi[4], Daniel J. Finley[4], Bradley E. Bernstein[2,3], Andrew A. Lane[1,2]

[1] Department of Medical Oncology, Dana-Farber Cancer Institute, Harvard Medical School, Boston, MA
[2] Broad Institute of Harvard and MIT, Cambridge, MA
[3] Department of Pathology, Massachusetts General Hospital, Harvard Medical School, Boston, MA
[4] Department of Cell Biology, Harvard Medical School, Boston, MA
[5] Center for Functional Cancer Epigenetics, Dana-Farber Cancer Institute, Boston, MA
[6] Laboratory of Metabolism, National Cancer Institute, Bethesda, MD
[7] Center for Regenerative Medicine, Massachusetts General Hospital, Boston, MA

Supplementary Figures 1-11 with legends

Supplementary Data 1-7 descriptions

Suppl Figure 1

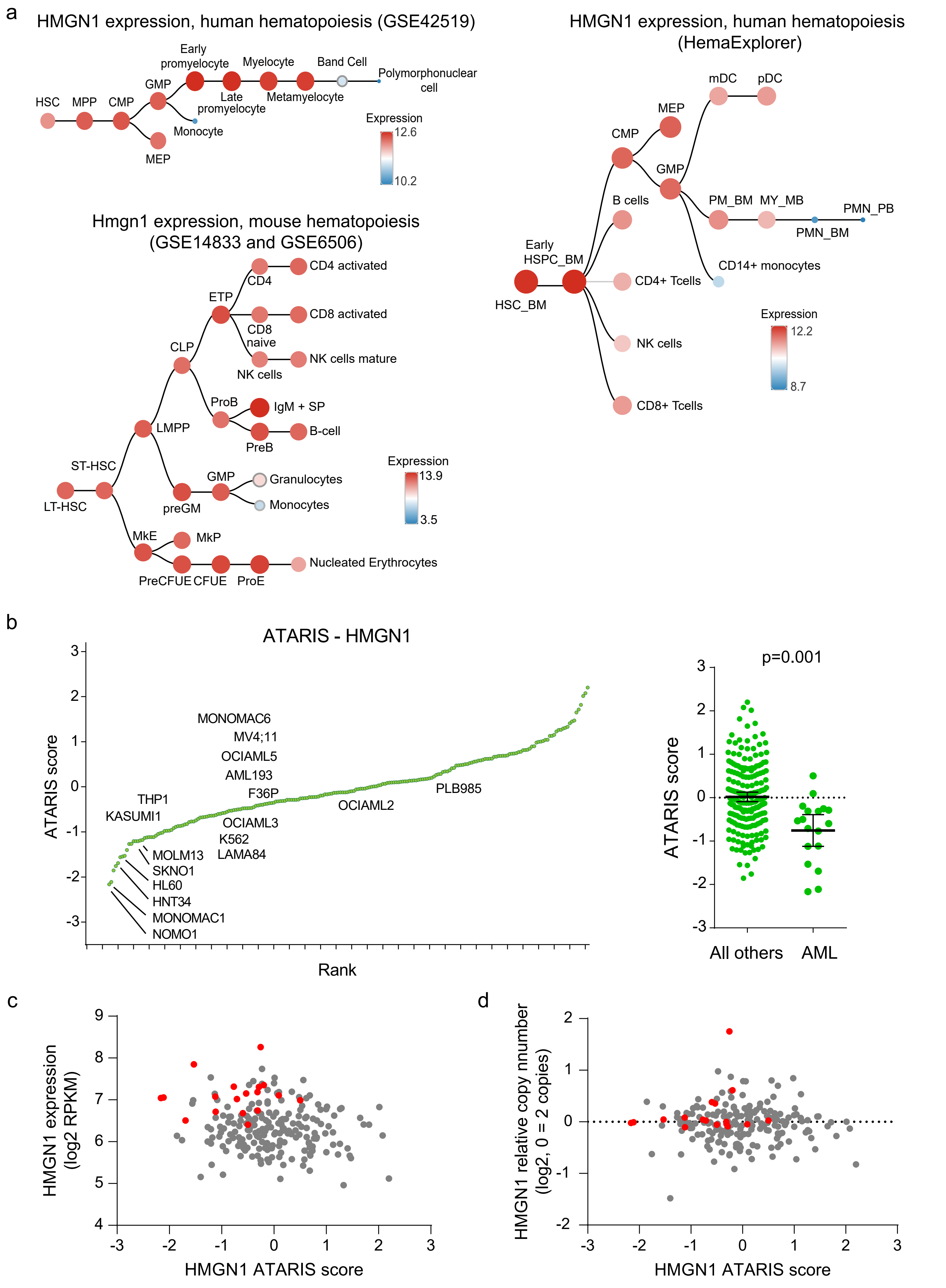

Supplementary Figure 1: HMGN1 decreases during myeloid differentiation and HMGN1 loss causes a selective disadvantage in AML cells.

a) Hierarchical hematopoietic tree (data visualization from BloodSpot[73]) showing higher *HMGN1* expression (red) in immature HSPCs compared to lower (blue) in mature, differentiated polymorphonuclear granulocytes (PMNs) and monocytes in three different datasets evaluating human and murine hematopoiesis. b) ATARIS profile for *HMGN1* in 216 cells lines from Project Achilles (dataset v2.4.3)[23]. Lower ATARIS score indicates selective disadvantage in that cell line compared to the group average. The left panel highlights the names of all AML-derived cell lines. The right panel compares ATARIS score for *HMGN1* in AML-derived cell lines (n=18) vs all others (n=198), data compared by 2-sided t test. Data are presented as mean values +/- SD. c) *HMGN1* expression and d) DNA copy number plotted vs ATARIS score for the Achilles cell lines. AML cells are indicated in red. Source data are provided as a Source Data file.

Suppl Figure 2

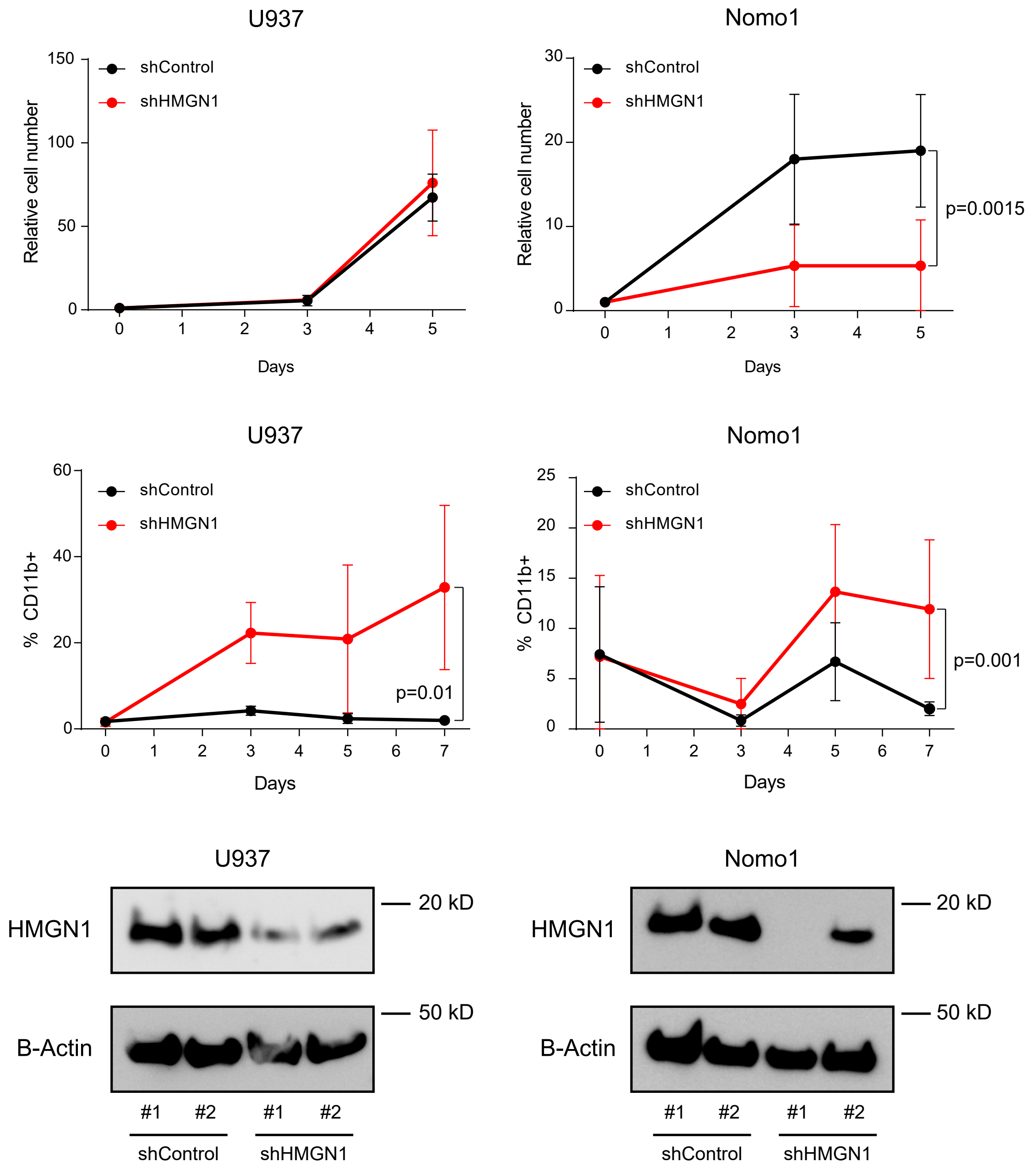

Supplementary Figure 2: HMGN1 loss impairs growth and/or promotes myeloid differentiation in AML cell lines.

Targeting *HMGN1* by CRISPR-Cas9 in the indicated AML cell lines was followed by analysis of proliferation and differentiation, compared to control sgRNAs, n=2 independent sgRNAs each for control and *HMGN1*, assessed as n=8 (control) or n=6 *HMGN1* biologically independent replicates for proliferation and n=4 biologically independent replicates for differentiation, genotypes compared by 2-sided t test. Data are presented as mean values +/- SD. HMGN1 expression levels after knockdown were assessed by western blot. Source data are provided as a Source Data file.

Suppl Figure 3

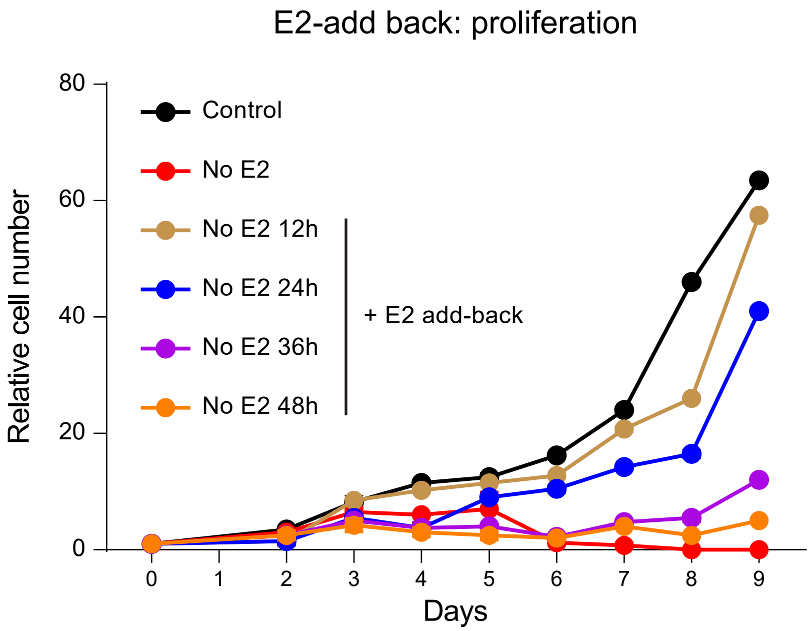

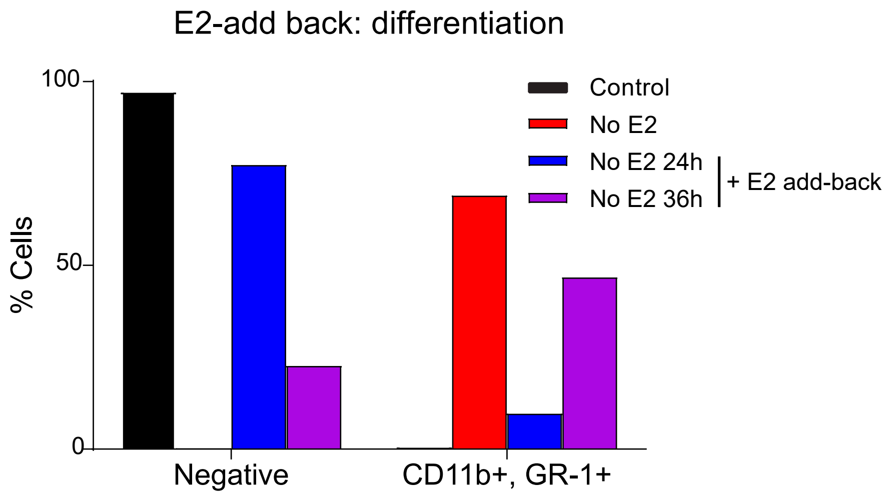

Supplementary Figure 3: Growth and differentiation of myeloid progenitors with re-introduction of estradiol at various times after withdrawal.

Analysis of proliferation (mean of n=2 biological replicates) and differentiation (measured as expression of CD11b and GR-1) of wild-type myeloid progenitors with add-back of E2 at the indicated time points. Source data are provided as a Source Data file.

Suppl Figure 4

a

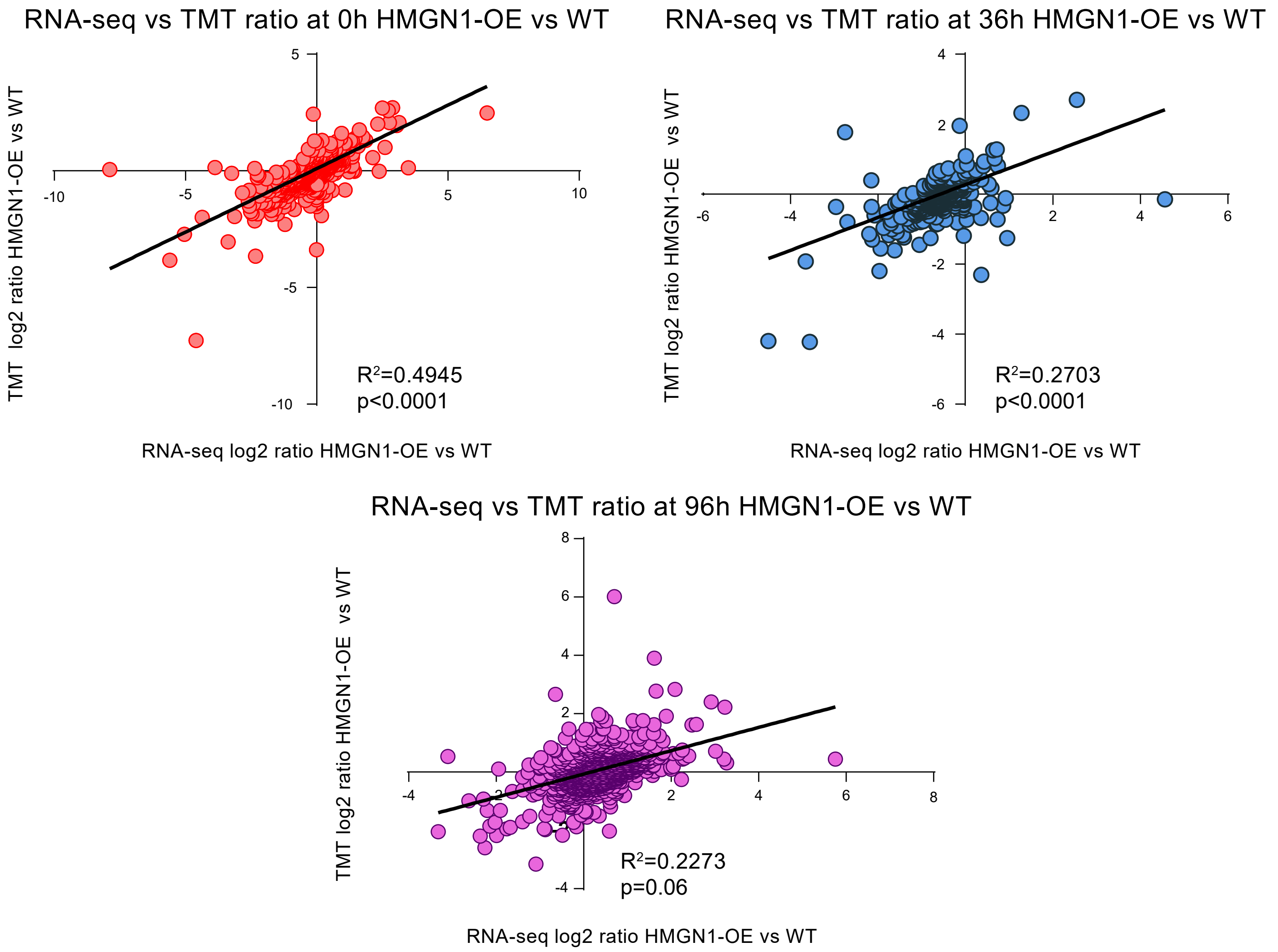

b

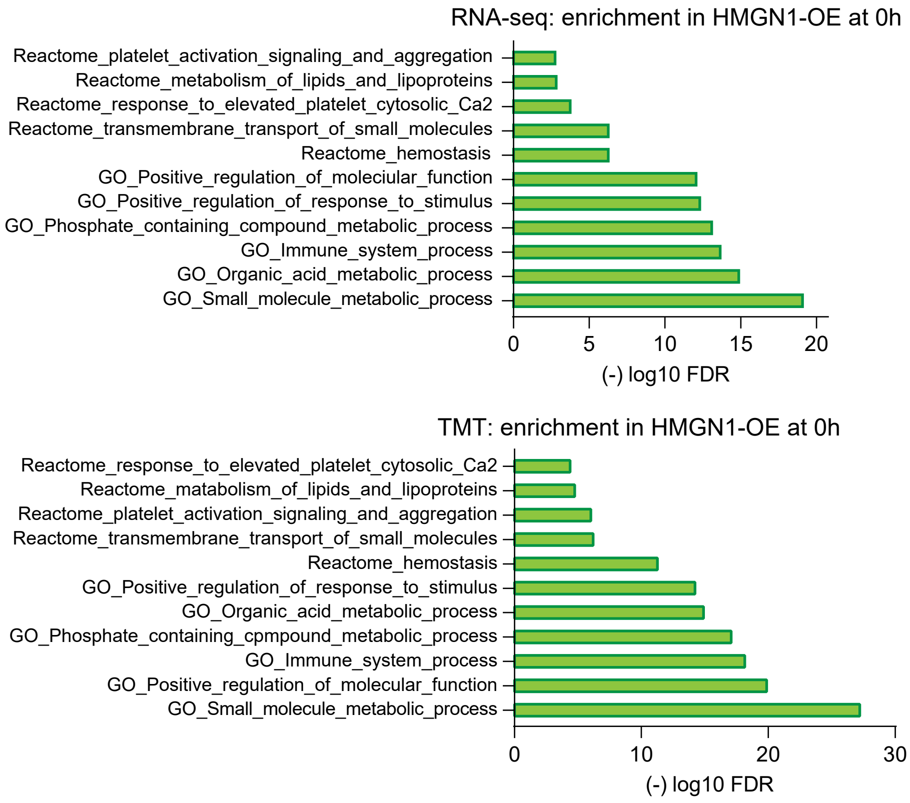

Supplementary Figure 4: RNA-seq and TMT proteomic data comparison.
a) Correlation by nonlinear regression between RNA-seq and TMT analysis represented as log2 ratio HMGN1-OE / wild-type at basal conditions and at 36h and 96h after withdrawal of E2. b) Reactome and Gene Ontology (GO) terms enriched in RNA-seq (top) and proteomic (bottom) analyses of HMGN1-OE compared to wild-type immature myeloid progenitors by gene set enrichment analysis (GSEA).

Suppl Figure 5

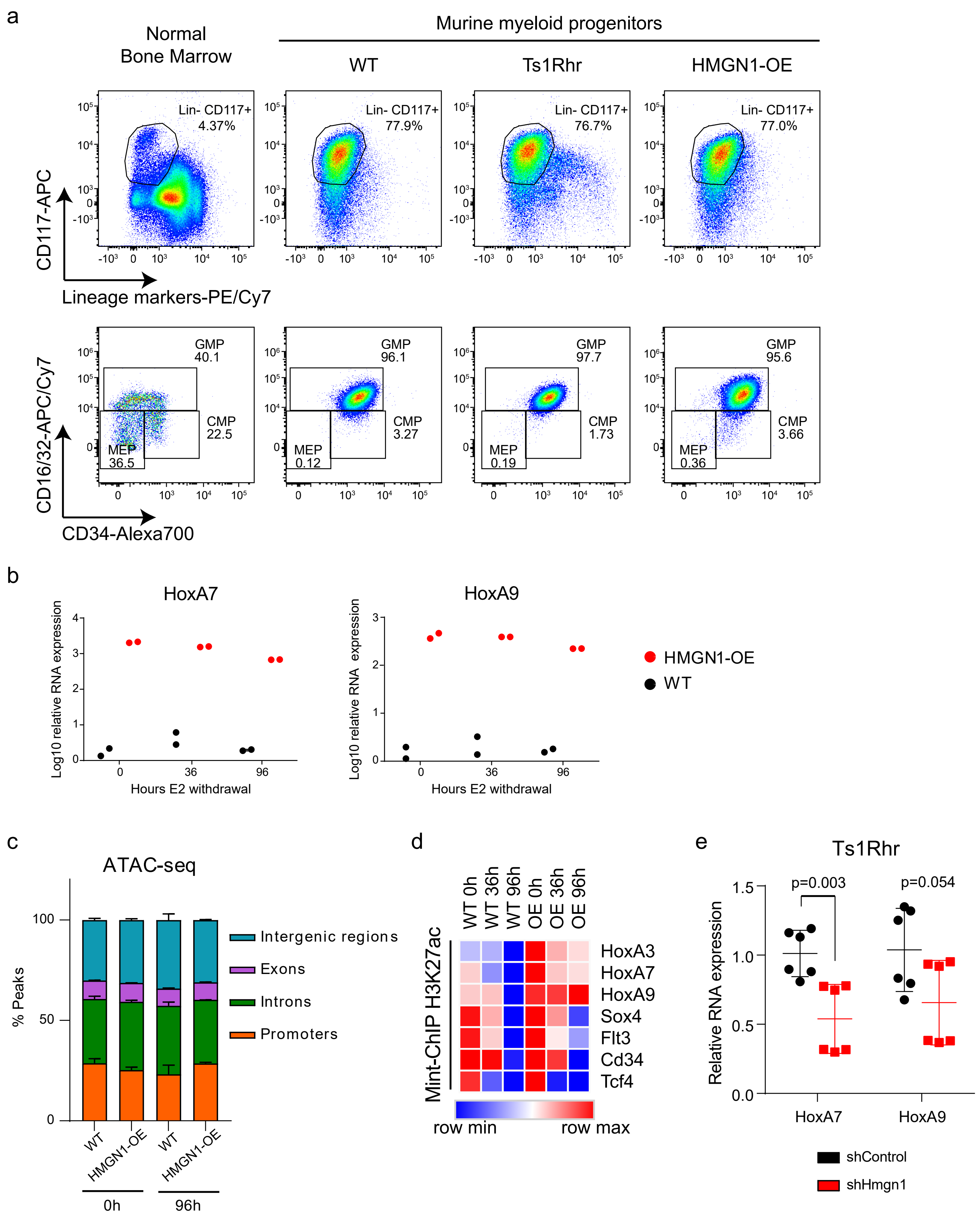

Supplementary Figure 5: Additional characterization of HMGN1-overexpressing myeloid progenitors and effects of modulating HMGN1 levels.

a) Surface marker expression in wild-type, Ts1Rhr, and HMGN1-OE murine myeloid progenitors compared to fresh murine bone marrow. b) Q-RT-PCR analysis of *HoxA7* and *HoxA9* in wild-type and HMGN1-OE immature murine progenitors during myeloid differentiation. n=2 biologically independent samples of each genotype, each measured in technical triplicate and displayed as the average of the technical triplicates. c) Distribution of ATAC-seq peaks in wild-type and HMGN1-OE myeloid progenitors at baseline and 96h after withdrawal of E2. d) Heatmap of H3K27ac levels measured by Mint-ChIP at the genomic loci of selected Hox genes and additional genes important in HSPCs. e) Q-RT-PCR of *HoxA7* and *HoxA9* expression in Ts1Rhr myeloid progenitors expressing shRNAs targeting *Hmgn1* or controls, conditions compared by 2-sided t test. n=3 biologically independent samples of each of two independent control shRNAs and Hmgn1 targeted shRNAs. Data are presented as mean values +/- SD. Source data are provided as a Source Data file.

Suppl Figure 6

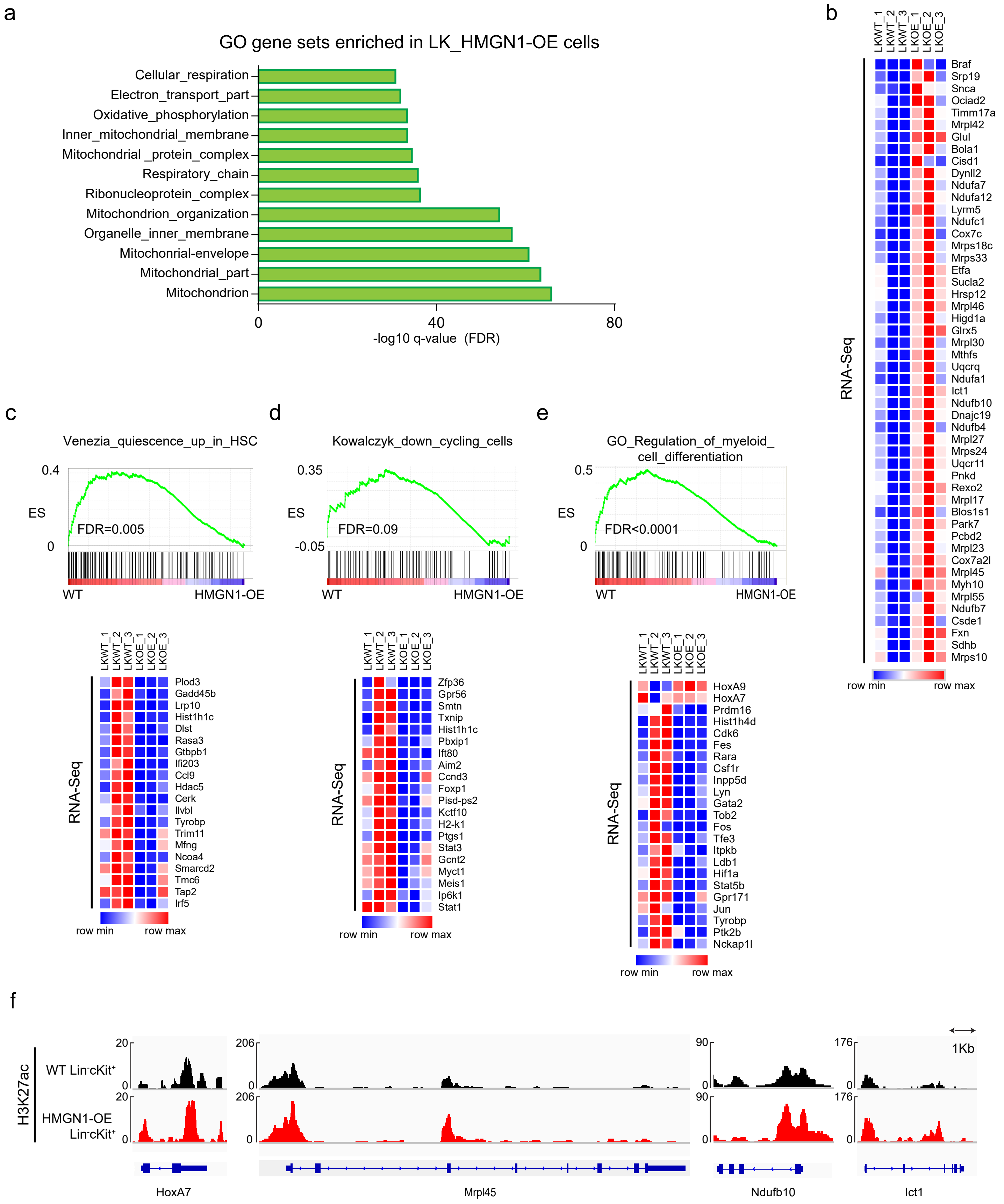

Supplementary Figure 6: HMGN1 overexpression is associated with quiescence and cell cycle-related gene expression changes in hematopoietic progenitors *in vivo*.

a) GSEA of GO terms enriched among genes showing fold change > 1.2 in HMGN1-OE vs wild-type LK HSPCs plotted as -log10 of q-value (false discovery rate, FDR). b) Heatmap of expression of the top 50 genes within the mitochondrial gene set GO_Mitochondrion enriched by GSEA in HMGN1-OE LK cells. c) GSEA plot and heatmap of the top 20 leading edge genes from the Venezia_quiescence_up_in_HSC gene set enriched in wild-type compared to HMGN1-OE LK cells. d) GSEA plot and heatmap of the top 20 leading edge genes from the Kowalczyk_down_cycling_cells gene set enriched in wild-type compared to HMGN1-OE LK cells. e) GSEA plot and heatmap of the top 20 leading edge genes from the GO_Regulation_of_myeloid_cell_differentiation gene set enriched in wild-type compared to HMGN1-OE LK cells, with *HoxA7* and *HoxA9* expression shown for comparison. f) Gene tracks of H3K27ac in loci of representative genes assayed by Mint-ChIP in LK HSPCs sorted from wild-type and HMGN1-OE bone marrow *in vivo*.

Suppl Figure 7

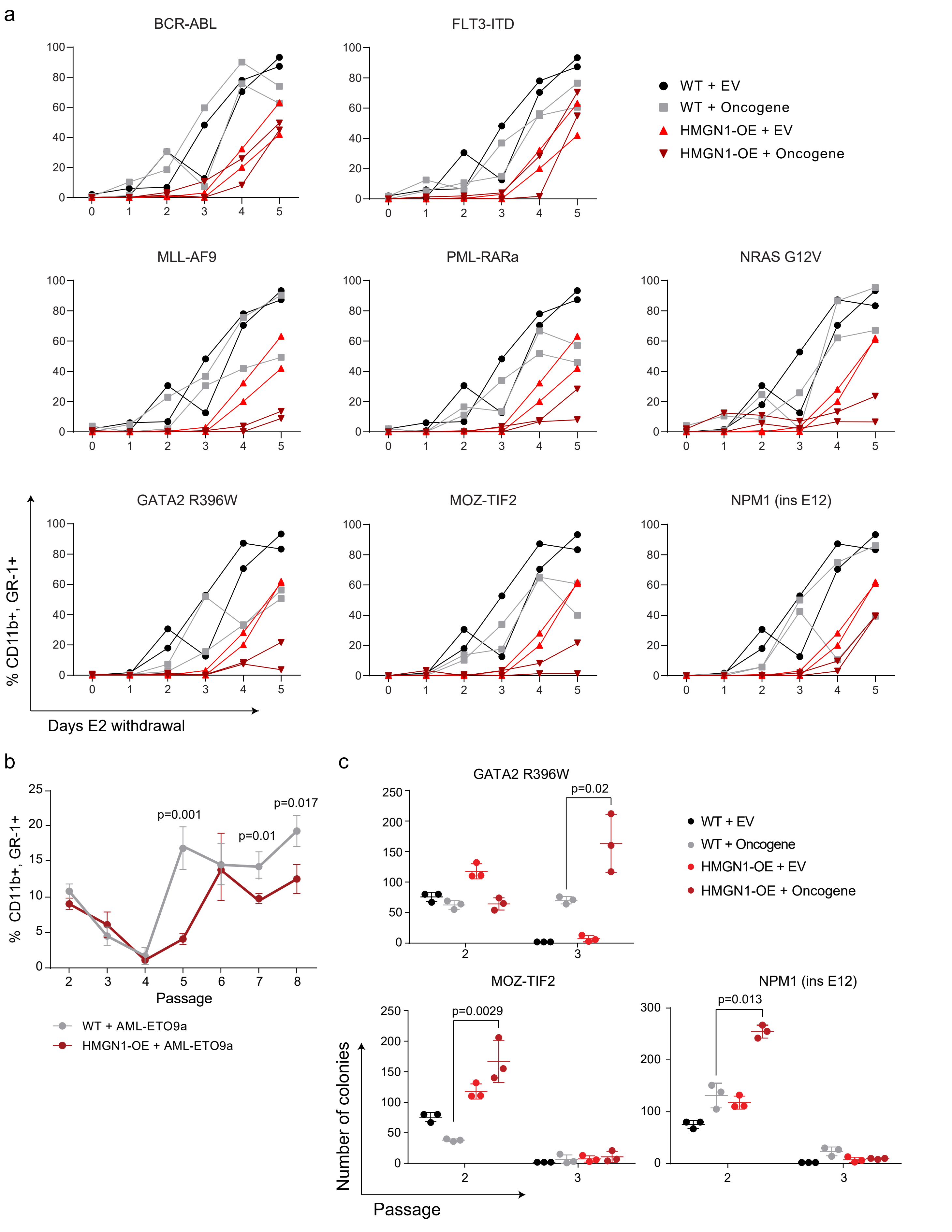

Supplementary Figure 7: Effect of leukemia oncogenes on differentiation and self-renewal in the setting of HMGN1 overexpression.

a) Analysis of myeloid differentiation (quantified by CD11b and GR-1 expression) in wild-type and HMGN1-OE murine myeloid progenitors transduced to express BCR-ABL, FLT3-ITD, MLL-AF9, PML-RARα, NRAS G12V, MOZ-TIF2, GATA2 R396W, or NPM1mut (exon 12 insertion). n=2 biologically independent cultures of each condition. b) Quantification of CD11b/GR-1-positive cells at each passage of the clonogenic assay shown in Figure 5b, n=3 biologically independent cultures. c) Serial replating of wild-type and HMGN1-OE myeloid colonies from murine myeloid progenitors transduced with GATA2 R396W, MOZ-TIF2, or NPM1mut oncogenes. In panel b and c, n=3 biologically independent cultures, genotypes compared by 2-sided t test. Data are presented as mean values +/- SD. Source data are provided as a Source Data file.

Suppl Figure 8

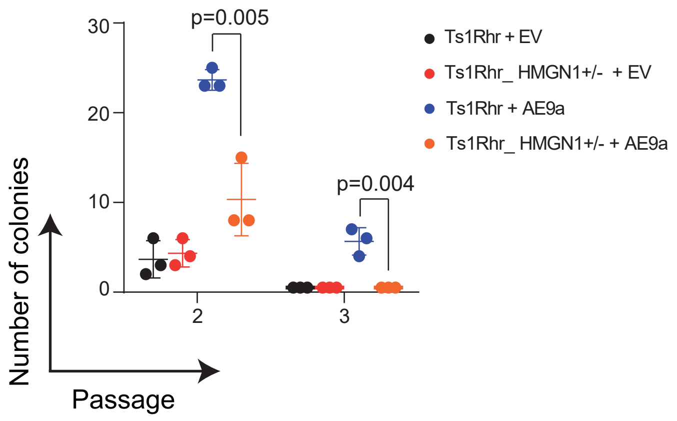

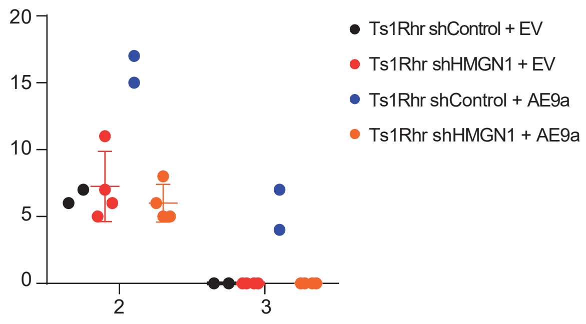

Supplementary Figure 8: Copy number and expression level of *Hmgn1* affect clonogenic capacity of Ts1Rhr myeloid progenitors.

Clonogenic assay of serial replating of Ts1Rhr myeloid progenitors with and without AML-ETO9a (AE9a) using two different approaches to restore *Hmgn1* levels. (Left) Ts1Rhr (3 copies of 31 genes) compared to Ts1Rhr_HMGN1+/- (2 copies of *Hmgn1* but 3 copies of the other 30 genes), each expressing AE9a or empty vector (EV). (Right) Ts1Rhr cells with *Hmgn1* or control shRNAs each expressing AE9a or EV. n=3 biologically independent samples at initial plating, data shown are those with measurable colonies at each indicated passage. Samples compared by 2-sided t test. Data are presented as mean values +/- SD. Source data are provided as a Source Data file.

Suppl Figure 9

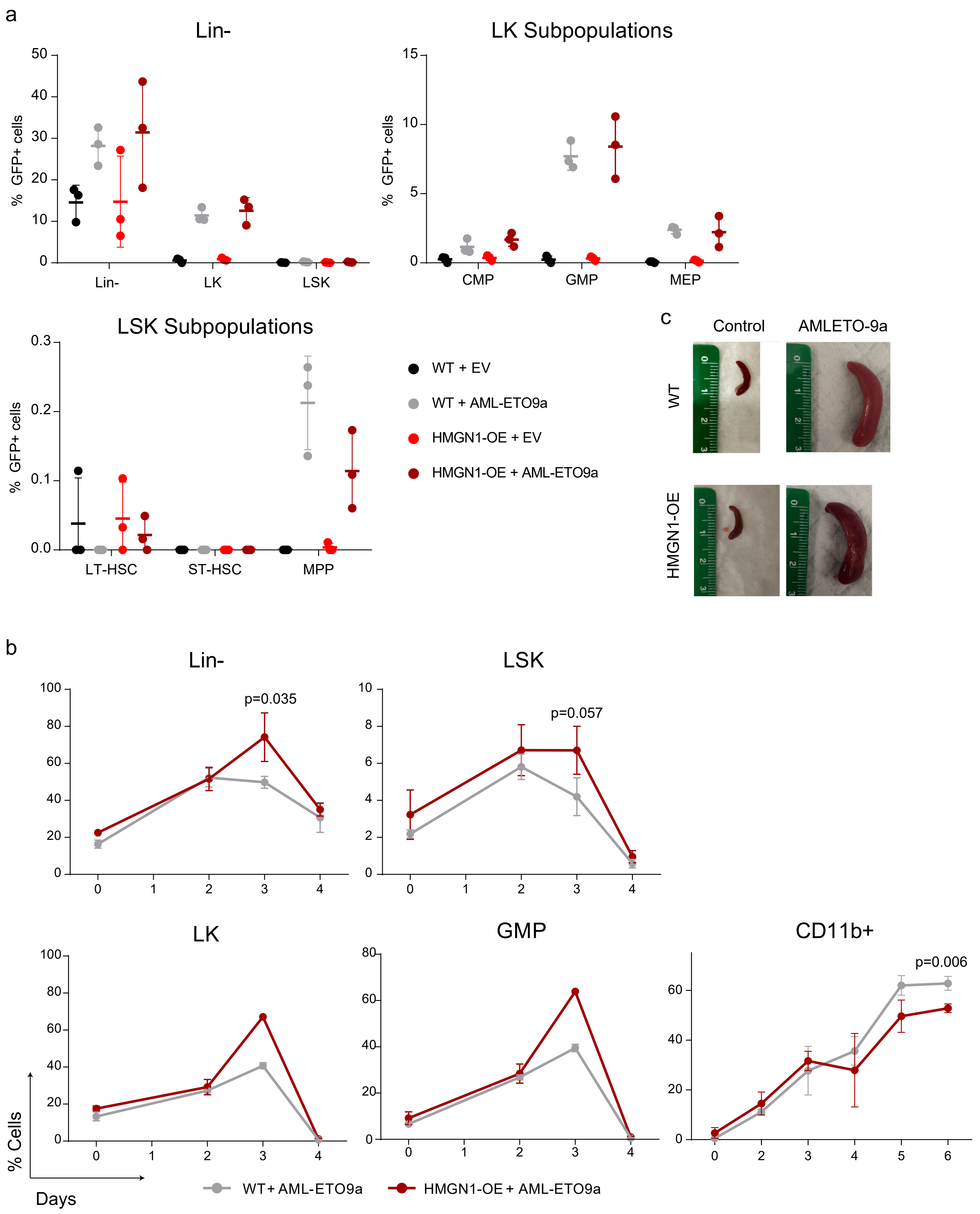

Supplementary Figure 9: *In vivo* analysis of HMGN1 overexpression combined with AML-ETO9a.

a) Analysis of hematopoietic subpopulations in bone marrow of mice sacrificed two months after transplantation with cells of the indicated genotypes transduced with the indicated retroviruses. b) Analysis of preleukemic hematopoietic subpopulations during *in vitro* liquid culture of CD117-enriched bone marrow cells harvested 2 months after transplantation of cells with the indicated genotypes. GMP are represented as percentage of LK population, others as percentage of all cells. In panel a and b, n=3 biologically independent replicates, genotypes compared by 2-sided t test. c) Representative images of spleens from AML-ETO9a-expressing wild-type or HMGN1-OE moribund mice after the primary transplant compared to normal control spleens from the same genotypes. Data are presented as mean values +/- SD. Source data are provided as a Source Data file.

Suppl Figure 10

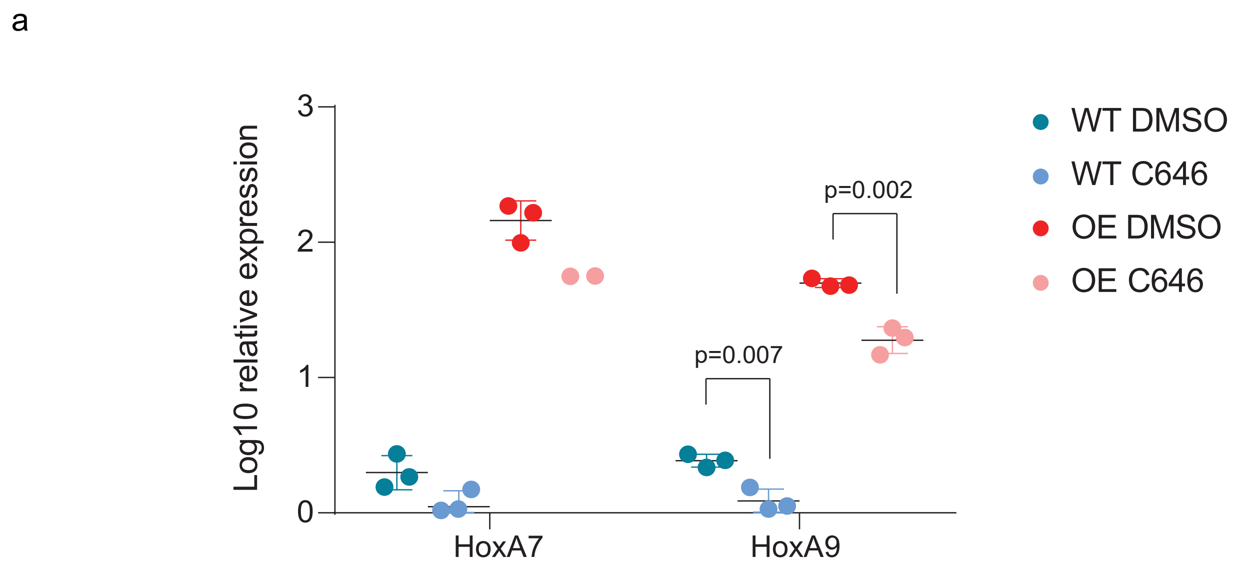

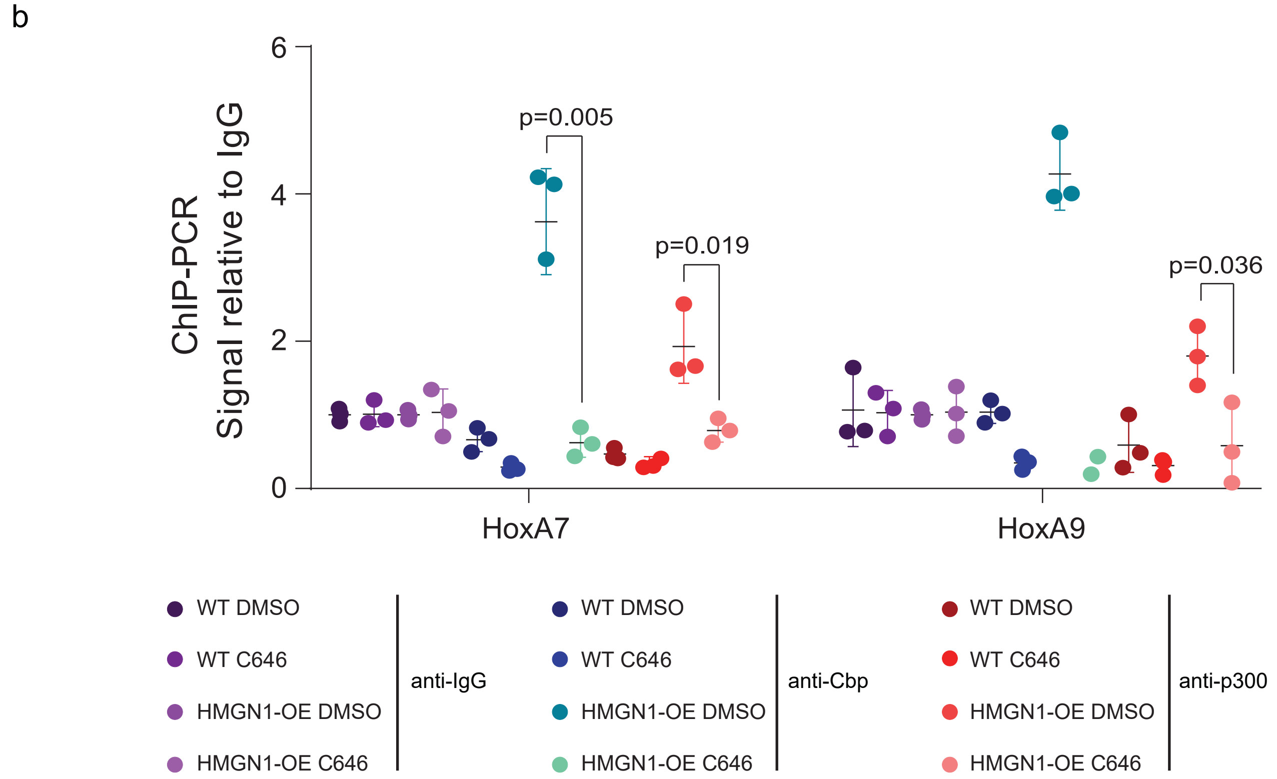

Supplementary Figure 10: Effect of C646 on HoxA gene expression and HAT binding at HoxA loci.

a) Q-RT-PCR analysis of *HoxA7* and *HoxA9* expression in wild-type and HMGN1-OE myeloid progenitors after C646 treatment for 96h in the absence of E2. n=3 biologically independent samples were plated, samples were assayed that had adequate RNA for RT-PCR. b) ChIP-PCR in the same cells after C646 treatment for relative p300 and Cbp binding at *HoxA7* and *HoxA9* loci. n=3 biologically independent samples were plated, samples were assayed that had sufficient quality cross-linked, sheared, and extracted DNA for ChIP-PCR. Samples compared by 2-sided t test. Data are presented as mean values +/- SD. Source data are provided as a Source Data file.

Suppl Figure 11

a

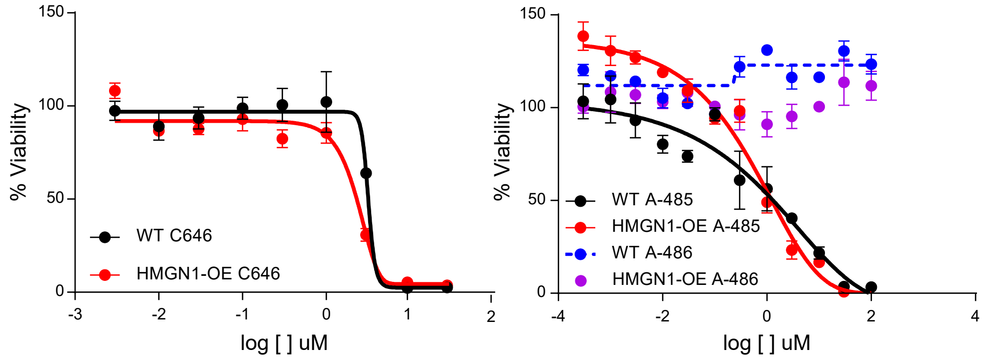

b

IC50 (uM)	WT		HMGN1-OE	
	IC25	IC50	IC25	IC50
GSKJ4	0.33	1	0.86	2
GSK126	2	6	2.3	7
BMS-3031411	10	30	7.6	26
Cbp30	1.2	3.6	1.1	3.3
iCbp112	3	9	3.7	11
Medica16	R	R	R	R
JQ-1	0.017	0.051	0.011	0.033

c

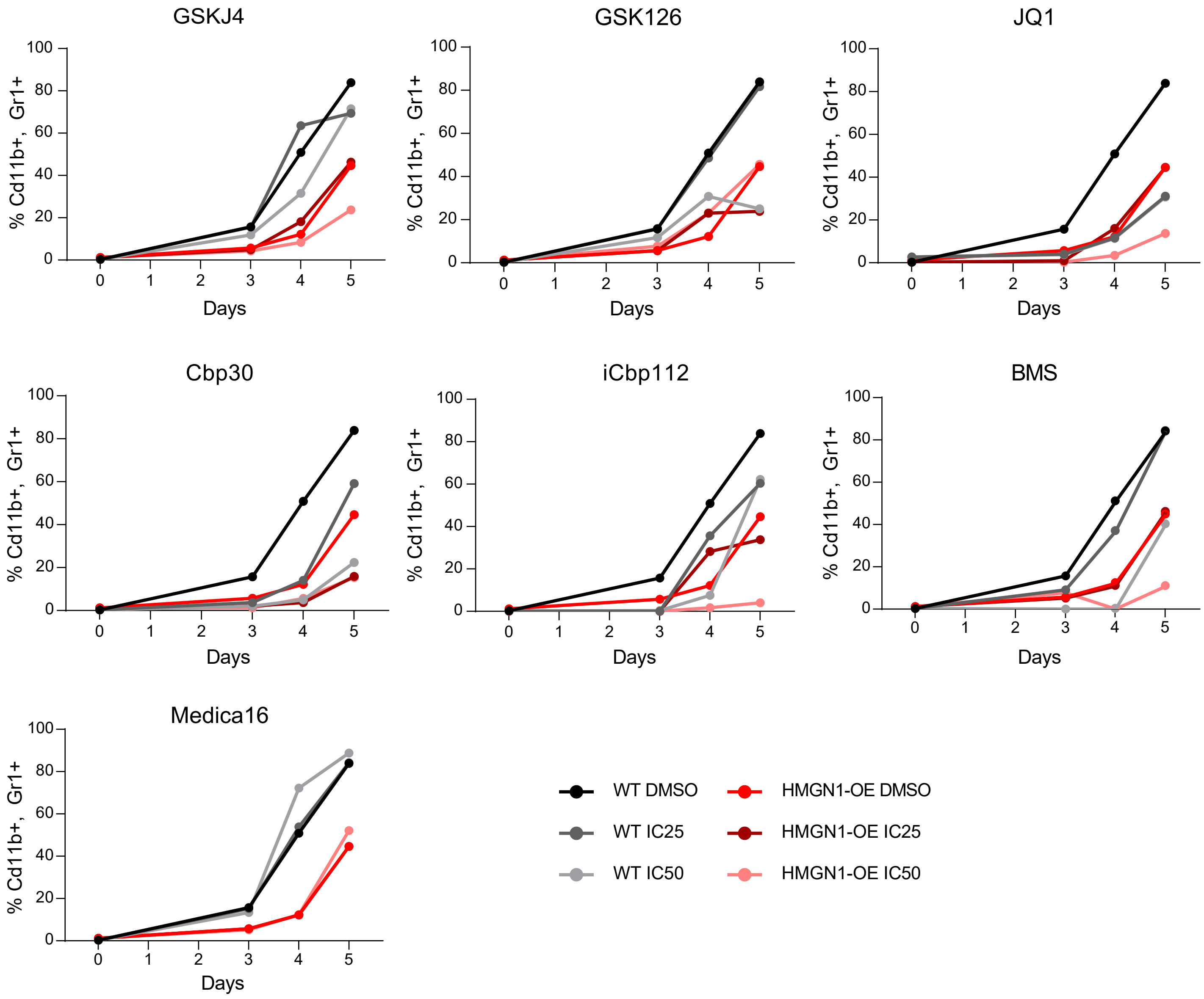

Supplementary Figure 11: Effect of small molecule treatment on myeloid differentiation in wild-type and HMGN1-OE cells.

a) Relative viability after treatment with C646 (left) and A-485 (right, compared to the inactive analog A-486) as measured by MTT in wild-type and HMGN1-OE cells. n=3 biologically independent samples per treatment. Data are presented as mean values +/- SD. b) IC25 and IC50 values (concentration resulting in 25% or 50% cell number compared to vehicle) of compounds targeting bromodomains (JQ1; Cbp30; iCbp112); ATP citrate lyase, ACLY (BMS-303141; Medica16); H3K27 demethylases (GSKJ4), and the H3K27 methyltransferase EZH2 (GSK126) in wild-type and HMGN1-OE myeloid progenitors calculated after 72h of drug treatment in absence of E2. c) Effect of compounds at their respective IC25 and IC50 on differentiation in wild-type and HMGN1-OE cells measured by flow cytometry for CD11b and GR-1 surface markers. Source data are provided as a Source Data file.
