# Supplementary material for: Chromatin accessibility promotes hematopoietic and leukemia stem cell activity
Source: Nat Commun. 2020 Mar 16;11:1406. doi: 10.1038/s41467-020-15221-z (PMC7076002; doi:10.1038/s41467-020-15221-z)
Supplement: Supplementary file 3 — Description of Additional Supplementary Files [file 41467_2020_15221_MOESM3_ESM.pdf]

Description of Additional Supplementary Files

Supplementary Data 1: Gene Set Brown_Myeloid _Cell_Development_Up. RNA-seq fold change (FC) of genes within the Brown_Myeloid_Cell_Development_Up gene set in HMGN1-OE compared to wild-type immature progenitors. Leading edge genes by GSEA are marked.

Supplementary Data 2: Gene Set Eppert_LSC_signature. RNA-seq fold change (FC) of genes within the Eppert_LSC_Signature gene set in HMGN1-OE compared to wild-type immature progenitors. Leading edge genes by GSEA are marked.

Supplementary Data 3: HoxA and HoxB family gene expression in myeloid progenitor cells. RNA-seq fold change (FC) of HoxA and HoxB family genes in HMGN1-OE compared to wild-type immature progenitors.

Supplementary Data 4: GO_Mitochondrion gene set in LK cells. RNA-seq fold change (FC) of genes within the GO_Mitochondrion gene set that are upregulated in HMGN1-OE compared to wild-type LK HSPCs harvested from the competitive bone marrow transplantation of CD45.1 wild-type vs CD45.2 HMGN1-OE cells.

Supplementary Data 5: Gene set Venezia_quiescence_up_in_HSC. RNA-seq fold change (FC) of genes within the Venezia_quiescence_up_in_HSC gene set in HMGN1-OE compared to wild-type LK HSPCs harvested from the competitive bone marrow transplantation of CD45.1 wild-type vs CD45.2 HMGN1-OE cells. Leading edge genes by GSEA are marked.

Supplementary Data 6: Gene set Kowalczyk_down_cycling_cells. RNA-seq fold change (FC) of genes within the Kowalczyk_down_cycling_cells gene set in HMGN1-OE compared to wild-type LK HSPCs harvested from the competitive bone marrow transplantation of CD45.1 wild-type vs CD45.2 HMGN1-OE cells. Leading edge genes by GSEA are marked.

Supplementary Data 7: Gene set GO_Regulation_of_myeloid_cell_differentiation. RNA-seq fold change (FC) of genes within the GO_Regulation_of_myeloid_cell_differentiation gene set in HMGN1-OE compared to wild-type LK HSPCs harvested from the competitive bone marrow transplantation of CD45.1 wild-type vs CD45.2 HMGN1-OE cells. Leading edge genes by GSEA are marked.
