# Supplementary material for: Chromatin accessibility promotes hematopoietic and leukemia stem cell activity
Source: Nat Commun. 2020 Mar 16;11:1406. doi: 10.1038/s41467-020-15221-z (PMC7076002; doi:10.1038/s41467-020-15221-z)
Supplement: Supplementary file 11 — Reporting Summary [file 41467_2020_15221_MOESM11_ESM.pdf]

nature research

Corresponding author(s): Andrew Lane

Last updated by author(s): Feb 4, 2020

Reporting Summary

Nature Research wishes to improve the reproducibility of the work that we publish. This form provides structure for consistency and transparency in reporting. For further information on Nature Research policies, see Authors & Referees and the Editorial Policy Checklist.

Statistics

For all statistical analyses, confirm that the following items are present in the figure legend, table legend, main text, or Methods section.

n/a	Confirmed	
☐	☒	The exact sample size (*n*) for each experimental group/condition, given as a discrete number and unit of measurement
☐	☒	A statement on whether measurements were taken from distinct samples or whether the same sample was measured repeatedly
☐	☒	The statistical test(s) used AND whether they are one- or two-sided<br>*Only common tests should be described solely by name; describe more complex techniques in the Methods section.*
☐	☒	A description of all covariates tested
☐	☒	A description of any assumptions or corrections, such as tests of normality and adjustment for multiple comparisons
☐	☒	A full description of the statistical parameters including central tendency (e.g. means) or other basic estimates (e.g. regression coefficient) AND variation (e.g. standard deviation) or associated estimates of uncertainty (e.g. confidence intervals)
☐	☒	For null hypothesis testing, the test statistic (e.g. *F*, *t*, *r*) with confidence intervals, effect sizes, degrees of freedom and *P* value noted<br>*Give P values as exact values whenever suitable.*
☒	☐	For Bayesian analysis, information on the choice of priors and Markov chain Monte Carlo settings
☒	☐	For hierarchical and complex designs, identification of the appropriate level for tests and full reporting of outcomes
☒	☐	Estimates of effect sizes (e.g. Cohen's *d*, Pearson's *r*), indicating how they were calculated

Our web collection on statistics for biologists contains articles on many of the points above.

Software and code

Policy information about availability of computer code

Data collection	No software was used.
Data analysis	GraphPad Prism 8. FlowJo X. Bowtie (version 0.12.2). GSEA (http://www.broadinstitute.org/gsea/) was performed using the Molecular Signatures Database (MSigDB) version 6.061. Heat maps visualized using Morpheus (http://software.broadinstitute.org/morpheus/). Gene tracks were visualized in the Integrated Genomics Viewer (IGV, Broad Institute). For ATAC-seq: ChiLin pipeline 2.0.0 for QC and preprocessing, Burrows-Wheeler Aligner (BWA) for read mapping, Model-based Analysis of ChIP-Seq (MACS) as a peak caller, and DESeq2 for differential peak analysis. Mint-Chip analysis https://www.protocols.io/view/mint-chip3-a-low-input-chip-seq-protocol-using-mul-wbefaje.

For manuscripts utilizing custom algorithms or software that are central to the research but not yet described in published literature, software must be made available to editors/reviewers. We strongly encourage code deposition in a community repository (e.g. GitHub). See the Nature Research guidelines for submitting code & software for further information.

Data

Policy information about availability of data

All manuscripts must include a data availability statement. This statement should provide the following information, where applicable:

- Accession codes, unique identifiers, or web links for publicly available datasets
- A list of figures that have associated raw data
- A description of any restrictions on data availability

Data Availability
RNA-seq, ATAC-seq, and Mint-ChIP data have been deposited in the GEO database under the SuperSeries accession code GSE143683 [https://www.ncbi.nlm.nih.gov/geo/query/acc.cgi?acc=GSE143683]. RNA-seq is in SubSeries GSE142473 [https://www.ncbi.nlm.nih.gov/geo/query/acc.cgi?acc=GSE142473], ATAC-seq is in SubSeries GSA143840 [https://www.ncbi.nlm.nih.gov/geo/query/acc.cgi?acc=GSE143840], and Mint-ChIP is in SubSeries GSE143682 [https://www.ncbi.nlm.nih.gov/geo/query/acc.cgi?acc=GSE143682]. Mass spectrometry proteomic data are available via ProteomeXchange with identifier PXD017054

[http://proteomecentral.proteomexchange.org/cgi/GetDataset?ID=PXD017054]. All the other data supporting the findings of this study are available within the article and its supplementary information files and from the corresponding author upon reasonable request. Source data are provided in a Source File. A reporting summary for this article is available as a Supplementary Information file.

Field-specific reporting

Please select the one below that is the best fit for your research. If you are not sure, read the appropriate sections before making your selection.

☒ Life sciences ☐ Behavioural & social sciences ☐ Ecological, evolutionary & environmental sciences

For a reference copy of the document with all sections, see nature.com/documents/nr-reporting-summary-flat.pdf

Life sciences study design

All studies must disclose on these points even when the disclosure is negative.

Sample size	Animal experiments were powered with sufficient mice included in each arm for ≥80% power with a 1-sided type I error rate of 0.05 to detect a 50% difference in survival between genotypes at a specified time point.
Data exclusions	No data were excluded.
Replication	Animal experiments were repeated independently with biologically independent host for transplant assays at least once. Replication was successful.
Randomization	Animals were randomly assigned to receive transplants by genotype or leukemia driver.
Blinding	Leukemia bearing mice were on tumor watch and were sacrificed at the first sign of physical extremis. The daily physical examination was performed by a team member who was blinded to the experimental arm.

Reporting for specific materials, systems and methods

We require information from authors about some types of materials, experimental systems and methods used in many studies. Here, indicate whether each material, system or method listed is relevant to your study. If you are not sure if a list item applies to your research, read the appropriate section before selecting a response.

Materials & experimental systems

n/a	Involved in the study	
☐	☒	Antibodies
☐	☒	Eukaryotic cell lines
☒	☐	Palaeontology
☐	☒	Animals and other organisms
☒	☐	Human research participants
☒	☐	Clinical data

Methods

n/a	Involved in the study	
☐	☒	ChIP-seq
☐	☒	Flow cytometry
☒	☐	MRI-based neuroimaging

Antibodies

Antibodies used

CD11b-PE Life Technologies #12-0112-81 Flow Cytometry; HMGN1 Life Technologies #A302363A Western-Blot;
GR1-FITC Fisher Scientific #509919 Flow Cytometry; B-Actin Sigma, #SAB5500001 Western-Blot; Anti-rabbit Alexa555 Life Technologies #A21428 Flow Cytometry; Histone H3 Cell Signaling Technology #9715 Western-Blot;
Lineage Cell Detection Cocktail-Biotin, mouse Miltenyi Biotech #130-092-613 Flow Cytometry; Histone H3 K27ac Cell Signaling Technology 8173 Western-Blot and Flow Cytometry;
Streptavidin-PE/Cy7 BioLegend # 405233 Flow Cytometry; Histone H3 K27ac Abcam, #ab4729 ChIP-PCR/WB/FC;
CD117-APC Fisher Scientific #BDB553356 Flow Cytometry; Histone H3 K27me3 Active Motif #61017 ChIP-PCR;
CD16/32 APC/Cy7 Fisher Scientific
#BDB560541 Flow Cytometry; CBP Cell Signaling Technology #7425 ChIP-PCR;
CD34-Alexa700 Fisher Scientific #BDB560518 Flow Cytometry; p300 Bethyl Laboratories #A300-358A-M ChIP-PCR;
CD150-Pac Blue BioLegend #115924 Flow Cytometry; Histone H3 Active Motif #39763 Mint-ChIP;
CD48-PE/Cy5 BioLegend #103420 Flow Cytometry; Histone H3 K27ac Active Motif #39133 Mint-ChIP;
CD45.1-FITC BioLegend #110706 Flow Cytometry; Histone H3 K27me3 Millipore #07-449 Mint-ChIP;
CD45.2-PERCP/Cy5.5 BioLegend #109828 Flow Cytometry ;
B220-PE BioLegend #103207 Flow Cytometry;
CD3-PB Fisher Scientific #BDB558214 Flow Cytometry ;
GR1-APC BioLegend #108412 Flow Cytometry ;
CD11b-APC/CY7 BioLegend-#101226 Flow Cytometry;

Validation | Validation with knockout and overexpression of HMGN1 in described experiments.

Eukaryotic cell lines

Policy information about cell lines

Cell line source(s) | Cell lines were from ATCC (U937) or DSMZ (Nomo1).

Authentication | STR profiling was performed every 2 years.

Mycoplasma contamination | Confirmed negative for mycoplasma contamination, tested every 6 months.

Commonly misidentified lines (See ICLAC register) | No commonly misidentified lines were used.

Animals and other organisms

Policy information about studies involving animals; ARRIVE guidelines recommended for reporting animal research

Laboratory animals | C57Bl6, Ts1Rhr, and HMGN1-OE transgenic mice were used, age was 6-12 weeks, both male and female mice were used, and were housed in specific pathogen-free laboratory mouse housing.

Wild animals | The study did not involve wild animals.

Field-collected samples | The study did not involve field-collected samples.

Ethics oversight | The animal protocol was approved by the Dana-Farber Animal Care and Use Committee.

Note that full information on the approval of the study protocol must also be provided in the manuscript.

ChIP-seq

Data deposition

☒ Confirm that both raw and final processed data have been deposited in a public database such as GEO.

☒ Confirm that you have deposited or provided access to graph files (e.g. BED files) for the called peaks.

Data access links
May remain private before publication. | Mint-ChIP is in GEO SubSeries GSE143682 [https://www.ncbi.nlm.nih.gov/geo/query/acc.cgi?acc=GSE143682]

Files in database submission |

ILMN_BC01_H3_15u_Ad_BC01_WT1_0h_exact_trim.fastq.gz
ILMN_BC01_H3_15u_Ad_BC02_WT1_36h_exact_trim.fastq.gz
ILMN_BC01_H3_15u_Ad_BC03_WT1_96h_exact_trim.fastq.gz
ILMN_BC01_H3_15u_Ad_BC04_WT2_0h_exact_trim.fastq.gz
ILMN_BC01_H3_15u_Ad_BC05_WT2_36h_exact_trim.fastq.gz
ILMN_BC01_H3_15u_Ad_BC06_WT2_96h_exact_trim.fastq.gz
ILMN_BC01_H3_15u_Ad_BC07_OE1_0h_exact_trim.fastq.gz
ILMN_BC01_H3_15u_Ad_BC08_OE1_36h_exact_trim.fastq.gz
ILMN_BC01_H3_15u_Ad_BC09_OE1_96h_exact_trim.fastq.gz
ILMN_BC01_H3_15u_Ad_BC10_OE2_0h_exact_trim.fastq.gz
ILMN_BC01_H3_15u_Ad_BC11_OE2_36h_exact_trim.fastq.gz
ILMN_BC01_H3_15u_Ad_BC12_OE2_96h_exact_trim.fastq.gz
ILMN_BC05_H3K27ac_15u_Ad_BC01_WT1_0h_exact_trim.fastq.gz
ILMN_BC05_H3K27ac_15u_Ad_BC02_WT1_36h_exact_trim.fastq.gz
ILMN_BC05_H3K27ac_15u_Ad_BC03_WT1_96h_exact_trim.fastq.gz
ILMN_BC05_H3K27ac_15u_Ad_BC04_WT2_0h_exact_trim.fastq.gz
ILMN_BC05_H3K27ac_15u_Ad_BC05_WT2_36h_exact_trim.fastq.gz
ILMN_BC05_H3K27ac_15u_Ad_BC06_WT2_96h_exact_trim.fastq.gz
ILMN_BC05_H3K27ac_15u_Ad_BC07_OE1_0h_exact_trim.fastq.gz
ILMN_BC05_H3K27ac_15u_Ad_BC08_OE1_36h_exact_trim.fastq.gz
ILMN_BC05_H3K27ac_15u_Ad_BC09_OE1_96h_exact_trim.fastq.gz
ILMN_BC05_H3K27ac_15u_Ad_BC10_OE2_0h_exact_trim.fastq.gz
ILMN_BC05_H3K27ac_15u_Ad_BC11_OE2_36h_exact_trim.fastq.gz
ILMN_BC05_H3K27ac_15u_Ad_BC12_OE2_96h_exact_trim.fastq.gz
ILMN_BC07_H3K27me3_15u_Ad_BC01_WT1_0h_exact_trim.fastq.gz
ILMN_BC07_H3K27me3_15u_Ad_BC02_WT1_36h_exact_trim.fastq.gz
ILMN_BC07_H3K27me3_15u_Ad_BC03_WT1_96h_exact_trim.fastq.gz
ILMN_BC07_H3K27me3_15u_Ad_BC04_WT2_0h_exact_trim.fastq.gz
ILMN_BC07_H3K27me3_15u_Ad_BC05_WT2_36h_exact_trim.fastq.gz
ILMN_BC07_H3K27me3_15u_Ad_BC06_WT2_96h_exact_trim.fastq.gz
ILMN_BC07_H3K27me3_15u_Ad_BC07_OE1_0h_exact_trim.fastq.gz

ILMN_BC07_H3K27me3_15u_Ad_BC08_OE1_36h_exact_trim.fastq.gz
ILMN_BC07_H3K27me3_15u_Ad_BC09_OE1_96h_exact_trim.fastq.gz
ILMN_BC07_H3K27me3_15u_Ad_BC10_OE2_0h_exact_trim.fastq.gz
ILMN_BC07_H3K27me3_15u_Ad_BC11_OE2_36h_exact_trim.fastq.gz
ILMN_BC07_H3K27me3_15u_Ad_BC12_OE2_96h_exact_trim.fastq.gz
ILMN_BC02_H3_60u_Ad_BC12_WT1_0h_exact_trim.fastq.gz
ILMN_BC02_H3_60u_Ad_BC11_WT1_36h_exact_trim.fastq.gz
ILMN_BC02_H3_60u_Ad_BC10_WT1_96h_exact_trim.fastq.gz
ILMN_BC02_H3_60u_Ad_BC09_WT2_0h_exact_trim.fastq.gz
ILMN_BC02_H3_60u_Ad_BC08_WT2_36h_exact_trim.fastq.gz
ILMN_BC02_H3_60u_Ad_BC07_WT2_96h_exact_trim.fastq.gz
ILMN_BC02_H3_60u_Ad_BC06_OE1_0h_exact_trim.fastq.gz
ILMN_BC02_H3_60u_Ad_BC05_OE1_36h_exact_trim.fastq.gz
ILMN_BC02_H3_60u_Ad_BC04_OE1_96h_exact_trim.fastq.gz
ILMN_BC02_H3_60u_Ad_BC03_OE2_0h_exact_trim.fastq.gz
ILMN_BC02_H3_60u_Ad_BC02_OE2_36h_exact_trim.fastq.gz
ILMN_BC02_H3_60u_Ad_BC01_OE2_96h_exact_trim.fastq.gz
ILMN_BC06_H3K27ac_60u_Ad_BC12_WT1_0h_exact_trim.fastq.gz
ILMN_BC06_H3K27ac_60u_Ad_BC11_WT1_36h_exact_trim.fastq.gz
ILMN_BC06_H3K27ac_60u_Ad_BC10_WT1_96h_exact_trim.fastq.gz
ILMN_BC06_H3K27ac_60u_Ad_BC09_WT2_0h_exact_trim.fastq.gz
ILMN_BC06_H3K27ac_60u_Ad_BC08_WT2_36h_exact_trim.fastq.gz
ILMN_BC06_H3K27ac_60u_Ad_BC07_WT2_96h_exact_trim.fastq.gz
ILMN_BC06_H3K27ac_60u_Ad_BC06_OE1_0h_exact_trim.fastq.gz
ILMN_BC06_H3K27ac_60u_Ad_BC05_OE1_36h_exact_trim.fastq.gz
ILMN_BC06_H3K27ac_60u_Ad_BC04_OE1_96h_exact_trim.fastq.gz
ILMN_BC06_H3K27ac_60u_Ad_BC03_OE2_0h_exact_trim.fastq.gz
ILMN_BC06_H3K27ac_60u_Ad_BC02_OE2_36h_exact_trim.fastq.gz
ILMN_BC06_H3K27ac_60u_Ad_BC01_OE2_96h_exact_trim.fastq.gz
ILMN_BC08_H3K27me3_60u_Ad_BC12_WT1_0h_exact_trim.fastq.gz
ILMN_BC08_H3K27me3_60u_Ad_BC11_WT1_36h_exact_trim.fastq.gz
ILMN_BC08_H3K27me3_60u_Ad_BC10_WT1_96h_exact_trim.fastq.gz
ILMN_BC08_H3K27me3_60u_Ad_BC09_WT2_0h_exact_trim.fastq.gz
ILMN_BC08_H3K27me3_60u_Ad_BC08_WT2_36h_exact_trim.fastq.gz
ILMN_BC08_H3K27me3_60u_Ad_BC07_WT2_96h_exact_trim.fastq.gz
ILMN_BC08_H3K27me3_60u_Ad_BC06_OE1_0h_exact_trim.fastq.gz
ILMN_BC08_H3K27me3_60u_Ad_BC05_OE1_36h_exact_trim.fastq.gz
ILMN_BC08_H3K27me3_60u_Ad_BC04_OE1_96h_exact_trim.fastq.gz
ILMN_BC08_H3K27me3_60u_Ad_BC03_OE2_0h_exact_trim.fastq.gz
ILMN_BC08_H3K27me3_60u_Ad_BC02_OE2_36h_exact_trim.fastq.gz
ILMN_BC08_H3K27me3_60u_Ad_BC01_OE2_96h_exact_trim.fastq.gz
ILMN_BC01_H3_15u_Ad_BC01_WT1_0h_exact_R2.fastq.gz
ILMN_BC01_H3_15u_Ad_BC02_WT1_36h_exact_R2.fastq.gz
ILMN_BC01_H3_15u_Ad_BC03_WT1_96h_exact_R2.fastq.gz
ILMN_BC01_H3_15u_Ad_BC04_WT2_0h_exact_R2.fastq.gz
ILMN_BC01_H3_15u_Ad_BC05_WT2_36h_exact_R2.fastq.gz
ILMN_BC01_H3_15u_Ad_BC06_WT2_96h_exact_R2.fastq.gz
ILMN_BC01_H3_15u_Ad_BC07_OE1_0h_exact_R2.fastq.gz
ILMN_BC01_H3_15u_Ad_BC08_OE1_36h_exact_R2.fastq.gz
ILMN_BC01_H3_15u_Ad_BC09_OE1_96h_exact_R2.fastq.gz
ILMN_BC01_H3_15u_Ad_BC10_OE2_0h_exact_R2.fastq.gz
ILMN_BC01_H3_15u_Ad_BC11_OE2_36h_exact_R2.fastq.gz
ILMN_BC01_H3_15u_Ad_BC12_OE2_96h_exact_R2.fastq.gz
ILMN_BC05_H3K27ac_15u_Ad_BC01_WT1_0h_exact_R2.fastq.gz
ILMN_BC05_H3K27ac_15u_Ad_BC02_WT1_36h_exact_R2.fastq.gz
ILMN_BC05_H3K27ac_15u_Ad_BC03_WT1_96h_exact_R2.fastq.gz
ILMN_BC05_H3K27ac_15u_Ad_BC04_WT2_0h_exact_R2.fastq.gz
ILMN_BC05_H3K27ac_15u_Ad_BC05_WT2_36h_exact_R2.fastq.gz
ILMN_BC05_H3K27ac_15u_Ad_BC06_WT2_96h_exact_R2.fastq.gz
ILMN_BC05_H3K27ac_15u_Ad_BC07_OE1_0h_exact_R2.fastq.gz
ILMN_BC05_H3K27ac_15u_Ad_BC08_OE1_36h_exact_R2.fastq.gz
ILMN_BC05_H3K27ac_15u_Ad_BC09_OE1_96h_exact_R2.fastq.gz
ILMN_BC05_H3K27ac_15u_Ad_BC10_OE2_0h_exact_R2.fastq.gz
ILMN_BC05_H3K27ac_15u_Ad_BC11_OE2_36h_exact_R2.fastq.gz
ILMN_BC05_H3K27ac_15u_Ad_BC12_OE2_96h_exact_R2.fastq.gz
ILMN_BC07_H3K27me3_15u_Ad_BC01_WT1_0h_exact_R2.fastq.gz
ILMN_BC07_H3K27me3_15u_Ad_BC02_WT1_36h_exact_R2.fastq.gz
ILMN_BC07_H3K27me3_15u_Ad_BC03_WT1_96h_exact_R2.fastq.gz
ILMN_BC07_H3K27me3_15u_Ad_BC04_WT2_0h_exact_R2.fastq.gz
ILMN_BC07_H3K27me3_15u_Ad_BC05_WT2_36h_exact_R2.fastq.gz
ILMN_BC07_H3K27me3_15u_Ad_BC06_WT2_96h_exact_R2.fastq.gz
ILMN_BC07_H3K27me3_15u_Ad_BC07_OE1_0h_exact_R2.fastq.gz
ILMN_BC07_H3K27me3_15u_Ad_BC08_OE1_36h_exact_R2.fastq.gz
ILMN_BC07_H3K27me3_15u_Ad_BC09_OE1_96h_exact_R2.fastq.gz
ILMN_BC07_H3K27me3_15u_Ad_BC10_OE2_0h_exact_R2.fastq.gz

ILMN_BC07_H3K27me3_15u_Ad_BC11_OE2_36h_exact_R2.fastq.gz
ILMN_BC07_H3K27me3_15u_Ad_BC12_OE2_96h_exact_R2.fastq.gz
ILMN_BC02_H3_60u_Ad_BC12_WT1_0h_exact_R2.fastq.gz
ILMN_BC02_H3_60u_Ad_BC11_WT1_36h_exact_R2.fastq.gz
ILMN_BC02_H3_60u_Ad_BC10_WT1_96h_exact_R2.fastq.gz
ILMN_BC02_H3_60u_Ad_BC09_WT2_0h_exact_R2.fastq.gz
ILMN_BC02_H3_60u_Ad_BC08_WT2_36h_exact_R2.fastq.gz
ILMN_BC02_H3_60u_Ad_BC07_WT2_96h_exact_R2.fastq.gz
ILMN_BC02_H3_60u_Ad_BC06_OE1_0h_exact_R2.fastq.gz
ILMN_BC02_H3_60u_Ad_BC05_OE1_36h_exact_R2.fastq.gz
ILMN_BC02_H3_60u_Ad_BC04_OE1_96h_exact_R2.fastq.gz
ILMN_BC02_H3_60u_Ad_BC03_OE2_0h_exact_R2.fastq.gz
ILMN_BC02_H3_60u_Ad_BC02_OE2_36h_exact_R2.fastq.gz
ILMN_BC02_H3_60u_Ad_BC01_OE2_96h_exact_R2.fastq.gz
ILMN_BC06_H3K27ac_60u_Ad_BC12_WT1_0h_exact_R2.fastq.gz
ILMN_BC06_H3K27ac_60u_Ad_BC11_WT1_36h_exact_R2.fastq.gz
ILMN_BC06_H3K27ac_60u_Ad_BC10_WT1_96h_exact_R2.fastq.gz
ILMN_BC06_H3K27ac_60u_Ad_BC09_WT2_0h_exact_R2.fastq.gz
ILMN_BC06_H3K27ac_60u_Ad_BC08_WT2_36h_exact_R2.fastq.gz
ILMN_BC06_H3K27ac_60u_Ad_BC07_WT2_96h_exact_R2.fastq.gz
ILMN_BC06_H3K27ac_60u_Ad_BC06_OE1_0h_exact_R2.fastq.gz
ILMN_BC06_H3K27ac_60u_Ad_BC05_OE1_36h_exact_R2.fastq.gz
ILMN_BC06_H3K27ac_60u_Ad_BC04_OE1_96h_exact_R2.fastq.gz
ILMN_BC06_H3K27ac_60u_Ad_BC03_OE2_0h_exact_R2.fastq.gz
ILMN_BC06_H3K27ac_60u_Ad_BC02_OE2_36h_exact_R2.fastq.gz
ILMN_BC06_H3K27ac_60u_Ad_BC01_OE2_96h_exact_R2.fastq.gz
ILMN_BC08_H3K27me3_60u_Ad_BC12_WT1_0h_exact_R2.fastq.gz
ILMN_BC08_H3K27me3_60u_Ad_BC11_WT1_36h_exact_R2.fastq.gz
ILMN_BC08_H3K27me3_60u_Ad_BC10_WT1_96h_exact_R2.fastq.gz
ILMN_BC08_H3K27me3_60u_Ad_BC09_WT2_0h_exact_R2.fastq.gz
ILMN_BC08_H3K27me3_60u_Ad_BC08_WT2_36h_exact_R2.fastq.gz
ILMN_BC08_H3K27me3_60u_Ad_BC07_WT2_96h_exact_R2.fastq.gz
ILMN_BC08_H3K27me3_60u_Ad_BC06_OE1_0h_exact_R2.fastq.gz
ILMN_BC08_H3K27me3_60u_Ad_BC05_OE1_36h_exact_R2.fastq.gz
ILMN_BC08_H3K27me3_60u_Ad_BC04_OE1_96h_exact_R2.fastq.gz
ILMN_BC08_H3K27me3_60u_Ad_BC03_OE2_0h_exact_R2.fastq.gz
ILMN_BC08_H3K27me3_60u_Ad_BC02_OE2_36h_exact_R2.fastq.gz
ILMN_BC08_H3K27me3_60u_Ad_BC01_OE2_96h_exact_R2.fastq.gz

Genome browser session (e.g. UCSC)
Final submission

Methodology

Replicates
All chip-seq was performed with 2 or 3 biological replicates.

Sequencing depth
10M paired-end reads per sample. On average, 96% of reads aligned to mm9, 32% of reads were duplicate.

Antibodies
Histone H3 Active Motif #39763 Mint-ChIP;
Histone H3 K27ac Active Motif #39133 Mint-ChIP;
Histone H3 K27me3 Millipore #07-449 Mint-ChIP;

Peak calling parameters
Peaks were called using Homer with the following parameters:
findPeaks <H3K27ac_sample> -region -size 1000 -minDist 2000 -C 0 -L 50 -o auto -i <H3_sample>
findPeaks <H3K27me3_sample> -region -size 5000 -minDist 15000 -C 0 -L 0 -o auto -i <H3_sample>

Data quality
These parameters require each putative peak to have 4-fold more reads in the target experiment (H3K27ac or H3K27me3) than the control (H3), without Clonal Signal filtering and with custom Local Signal filtering (see Homer documentation).

Software
ChIP-seq was analyzed with BWA, Samtools, Picard, Homer, IGV, and RStudio (DESeq2, GenomicRanges). Details of the protocol are described at https://www.protocols.io/view/mint-chip3-a-low-input-chip-seq-protocol-using-mul-wbefaje.

Flow Cytometry

Plots

Confirm that:

☒ The axis labels state the marker and fluorochrome used (e.g. CD4-FITC).

☒ The axis scales are clearly visible. Include numbers along axes only for bottom left plot of group (a 'group' is an analysis of identical markers).

☒ All plots are contour plots with outliers or pseudocolor plots.

☒ A numerical value for number of cells or percentage (with statistics) is provided.

Methodology

Sample preparation	Bone marrow was harvested by flushing tibias and fibulas from mice.
Instrument	CytoFlex flow cytometer (Beckman Coulter), FACS Canto II, and FACS Aria II SORP
Software	FlowJo version X
Cell population abundance	Post-sort fractions were >95% pure and verified by secondary flow cytometry.
Gating strategy	FSC/SSC was the initial gating, then FSC-H/FSC-W for singlets, then cut offs for gating were based on comparing unstained, single stains, and fluorescence minus one strategies.

☒ Tick this box to confirm that a figure exemplifying the gating strategy is provided in the Supplementary Information.
